# Effects of osthole on osteoporotic rats: a systematic review and meta-analysis

**DOI:** 10.1080/13880209.2022.2110267

**Published:** 2022-08-18

**Authors:** Bin Wu, Xiu-Fang Zhu, Xiao-Qiang Yang, Wei-Yi Wang, Jian-Hua Lu

**Affiliations:** a The First Clinical Medical College, Zhejiang Chinese Medical University, Hangzhou, China; b Department of Pneumology, The First Affiliated Hospital of Zhejiang Chinese Medical University, Hangzhou, China; c Department of Orthopaedics, The First Affiliated Hospital of Zhejiang Chinese Medical University, Hangzhou, China

**Keywords:** *Cnidium monnieri*, osteoporosis, bone mineral density, animal experiments

## Abstract

**Context:**

*Cnidium monnieri* Cusson (Apiaceae) has been used in traditional Asian medicine for thousands of years. Recent studies showed its active compound, osthole, had a good effect on osteoporosis. But there was no comprehensive analysis.

**Objective:**

This meta-analysis evaluates the effects of osthole on osteoporotic rats and provides a basis for future clinical studies.

**Methods:**

Chinese and English language databases (e.g., PubMed, Web of Science, Cochrane Library, Google Scholar, Embase, China National Knowledge Infrastructure, Wanfang Data Knowledge Service Platform, Weipu Chinese Sci-tech periodical full-text database, and Chinese BioMedical Literature Database) were searched from their establishment to February 2021. The effects of osthole on bone mineral density, osteoclast proliferation, and bone metabolism markers were compared with the effects of control treatments.

**Results:**

To our knowledge, this is the first meta-analysis to evaluate osthole for the treatment of osteoporosis in rats. We included 13 randomized controlled studies conducted on osteoporotic rats. Osthole increased bone mineral density (standardized mean difference [SMD] = 3.08, 95% confidence interval [CI] = 2.08–4.09), the subgroup analysis showed that BMD significantly increased among rats in osthole <10 mg/kg/day and duration of osthole treatment >2 months. Osthole improved histomorphometric parameters and biomechanical parameters, also inhibited osteoclast proliferation and bone metabolism.

**Conclusions:**

Osthole is an effective treatment for osteoporosis. It can promote bone formation and inhibit bone absorption.

## Introduction

Osteoporosis is a systemic disease characterized by low bone mass and damage to bone microstructure. Because of advancing age and hormonal changes, postmenopausal women and elderly men experience a gradual decline in bone mass, leading to osteoporosis. Osteoporosis affects over 10 million Americans over 50, and with the expected growth of the ageing population this number is likely to increase in the future (Keshishi et al. 2021). The prevalence of osteoporosis among adults 40 years or older was 5.0% among men and 20.6% among women in the Chinese population (Wang et al. 2021). Osteoporotic bones are fragile and have an increased risk of fracture following minimal trauma; fracture may occur without trauma in some individuals (Cosman et al. 2014). Patients with osteoporotic fractures have a poor quality of life. Common fragility fracture sites being found in the hip, spine and wrist. Hip fractures are the most serious, since 20% more people die than expected for age within the first year (Fuggle et al. 2019; Guzon-Illescas et al. 2019). Worldwide, osteoporosis causes more than 9 million fractures a year, meaning there is an osteoporotic fracture every 3 sec (Borgström et al. 2020), such as vertebral compression fracture, distal radius fracture and femoral neck fracture, so they pose substantial economic and social burdens. The annual economic cost of fragility fractures in Europe was estimated at €37 billion (Hernlund et al. 2013). By 2050, the annual costs from osteoporotic fractures in China are expected to be $25.43 billion (Si et al. 2015).

Current drugs for osteoporosis could inhibit bone resorption and promote bone formation, leading to an increase in bone mass and bone mineral density (BMD), decrease in bone loss, and protection against fragility fractures (Yuan et al. 2019). However, there are concerns regarding the adverse effects of these drugs, including dizziness, leg cramps (Minisola et al. 2019), jaw osteonecrosis (Khan et al. 2015), increased risk of malignant tumours (Li et al. 2020), and impaired gastrointestinal and renal functions (Cosman et al. 2014). Therefore, newer drugs with better safety profiles and fewer side effects are needed to treat osteoporosis.

*Cnidium monnieri* Cusson (Apiaceae) has been used in traditional Asian medicine for thousands of years as a treatment for impotence, vulvar itching, and eczema. *C. monnieri* warms kidneys, strengthens *yang*, reduces dampness, and dispels wind. That means it can replenish *yang qi* in the kidneys, remove the dampness and wind pathogenic *qi* that causes diseases in human body. About 56 chemical structures have been identified in *C. monnieri*, which can be categorized as coumarins, volatile constituents, chromones, liposoluble compounds, monoterpenoid glucosides, terpenoids, and other compounds (Sun et al. 2020). In recent years, many investigators have evaluated *C. monnieri* and its active compound, osthole, for the treatment of osteoporosis (Zhang et al. 2017; Ma et al. 2019; Yu et al. 2020; Jin et al. 2021). Experiments conducted *in vitro* and *in vivo* have shown that osthole inhibits bone resorption, promotes bone formation, and increases bone mass.

We conducted this meta-analysis because there was significant heterogeneity in the outcomes assessed by previous studies. By performing this meta-analysis, we provide a basis for future clinical trials involving osthole in the treatment of osteoporosis.

## Materials and methods

This meta-analysis was performed in accordance with the recommendations of the Cochrane Handbook for Systematic Reviews of Interventions and followed the Preferred Reporting Items for Systematic Reviews and Meta-analysis (PRISMA) guidelines (Moher et al. 2009).

### Literature search

We searched Chinese and English language databases, including PubMed, Web of Science, Cochrane Library, Google Scholar, Embase, China National Knowledge Infrastructure, Wanfang Data Knowledge Service Platform, Weipu Chinese Sci-tech periodical full-text database, and Chinese BioMedical Literature Database. The literature search was performed independently by two authors. Keywords and Medical Subject Headings (MeSH) terms were used for searching databases. We searched the databases from their establishment to February 2021 using combinations of the following terms: osteoporosis, osteoporosis, postmenopausal, osteoporosis, senile, osteoporosis, age-related, osthole, *Fructus Cnidii, Cnidium monnieri,* animal models, and animal experiments.

### Inclusion and exclusion criteria

We selected randomized controlled trials that compared osthole with saline or placebo (vehicle-treatment) in osteoporotic rats. The research animals were osteoporotic rats (with or without ovariectomy), and the experiments were *in vivo*. Outcomes indicators were clear and corresponding data could be extracted. We excluded studies that included experimental rats with bone diseases other than osteoporosis. We also excluded studies that reported duplicate results, as well as studies published in the form of conference reports, reviews, editorials, or letters.

### Selection of studies

Following the exclusion of duplicate reports, two authors independently reviewed the remaining titles and abstracts to exclude studies that met the exclusion criteria. Full text articles of the remaining studies were reviewed to determine their eligibility for inclusion. Discrepancies between the selections by the two authors were resolved through discussion or by consulting a third author.

### Data extraction

Two authors independently extracted the following data from the included studies: first author; year of publication; method of osteoporosis induction; sample size; interventions; methods of administration; duration of study; and means, standard deviations (SDs), and standard errors of the outcomes. If data were presented in figures, the GetData software (http://getdata-graph-digitizer.com) was used to extract data from the figures.

### Risk of bias assessment

Two authors independently assessed the risk of bias using the tool developed by the Systematic Review Centre for Laboratory Animal Experimentation (SYRCLE) (Hooijmans et al. 2014). This tool consists of 10 items that assess the risk of bias in six domains: selection, performance, detection, attrition, reporting, and other biases. The risk of bias in each domain is rated as low, high, or unclear. The SYRCLE tool enlists criteria for low risk of bias in all domains. Studies that meet these criteria have a low risk of bias, studies that do not meet these criteria have a high risk of bias, and studies with an unclear description have an unclear risk of bias. Discrepancies were resolved through discussion or by consulting a third author.

### Statistical analysis

The means and SDs of continuous variables were recorded in Microsoft Excel (Microsoft Corp., Redmond, WA, USA). Heterogeneity among the studies was evaluated using the heterogeneity index I^2^. A random-effects model was used if significant heterogeneity was present (I^2^ > 50%); a fixed-effects model was used if no significant heterogeneity was present (I^2^ < 50%). Sources of heterogeneity were evaluated using subgroup and sensitivity analyses. Subgroup analyses were performed according to study type. Sensitivity analysis was carried out using the leave-one-out approach to determine the robustness of outcomes data. Publication bias was assessed using funnel plots and Egger’s test; *p*-values > 0.05 suggested that publication bias was absent. Review Manager (RevMan; version 5.2; Cochrane Collaboration, Oxford, UK) and Stata software (version 12.0; StataCorp, LLC, College Station, TX, USA) were used to analyse the data.

## Results

### Search results

**Figure 1.**
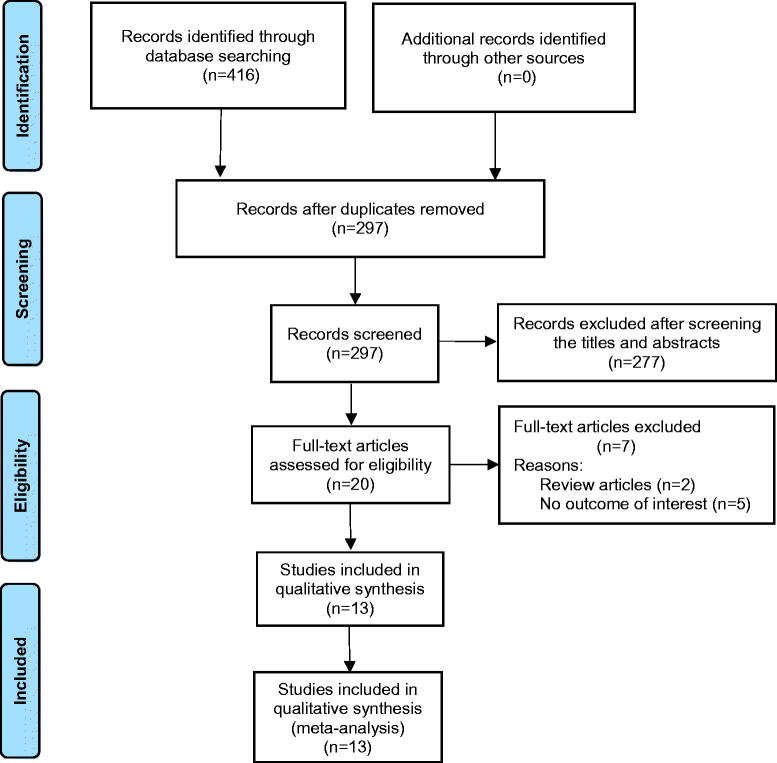
PRISMA flow chart of study selection. PRISMA, Preferred Reporting Items for Systematic Reviews and Meta-analysis.

The initial search identified 416 articles. After the exclusion of duplicate articles (119), 277 articles were excluded because their titles and abstracts did not meet the selection criteria. The full text versions of the remaining 20 articles were reviewed, and 7 articles were excluded because they were either review articles (2) or did not report relevant outcomes (5). The remaining 13 randomized controlled studies were included in this meta-analysis. Figure 1 depicts the process of article selection.

### Characteristics of included studies

The main characteristics of the included studies are summarized in Table 1. Thirteen randomized controlled studies of rats, published between 1996 and 2021, were included in this meta-analysis. Nine studies induced osteoporosis by bilateral ovariectomy; the remaining four studies induced osteoporosis by simulated microgravity, 5/6 nephrectomy, hormone administration, or senility. Most studies administered intragastric or intraperitoneal osthole to rats in the experimental group; one study administered intramuscular dexamethasone and osthole oral gavage. The study duration ranged from 4 to 12 weeks.

**Table 1.** Characteristics of the included studies.

First author	Induction of osteoporosis	Sample size		Intervention		Methods of administration	Duration of study
(year)		Osthole	Control	Osthole	Control		
Feng et al. 2018	Simulated microgravity	8	8	10 mg/kg/day	Equivalent normal saline	Intragastric	4 weeks
Guo et al. 2020	Ovariectomy	5	5	10 mg/kg/day	Equivalent normal saline	Intragastric	12 weeks
Jin et al. 2021	Senility	6	6	5 mg/kg/day	Vehicle (corn oil)	Intraperitoneal	4 weeks
Li et al. 1996	Ovariectomy	6	7	6.7 mg/kg, 6 days/week	Equivalent normal saline	Intragastric	12 weeks
Li et al. 2002	Ovariectomy	6	6	9 mg/kg, 5 days/week	Solvent vehicle	Oral gavage	4 weeks
Li et al. 2016	Nephrectomy	6	6	5 mg/kg/day	Vehicle (corn oil)	Intraperitoneal	2 months
Liu et al. 2020	Ovariectomy	10	10	10 mg/kg/day	Equivalent normal saline	Oral gavage	12 weeks
Sun et al. 2020	Ovariectomy	8	8	10 mg/kg/day	Equivalent normal saline	Intragastric	12 weeks
Tang et al. 2006	Hormone	8	8	10 mg/kg/day	Dexamethasone, 1 mg/kg, 2days/week	Intragastric	8 weeks
Tang et al. 2010	Ovariectomy	10	10	100 mg/kg/day	Vehicle control	Oral gavage	8 weeks
Wang et al. 2020	Ovariectomy	8	8	10 mg/kg/day	Equivalent normal saline	Oral gavage	12 weeks
Zhao et al. 2015	Ovariectomy	10	10	100 mg/kg/day	Equivalent normal saline	Intragastric	30 days
Zhao et al. 2018	Ovariectomy	6	6	10 mg/kg/day	Equivalent normal saline	Intragastric	3 months

### Risk of bias

Table 2 summarizes the results of risk of bias assessment using SYRCLE's risk of bias tool for animal studies. None of the included studies described the methods for randomization or randomized evaluation. Allocation concealment and blinding were unclear for all studies. Two studies housed the rats randomly. The median risk of bias score was 3 (range, 2–5).

**Table 2.** Quality of the included studies.

Publication	Year	(1)	(2)	(3)	(4)	(5)	(6)	(7)	(8)	(9)	(10)	Score
Feng et al.	2018		√						√	√	√	4
Guo et al.	2020		√							√	√	3
Jin et al.	2021								√	√	√	3
Li et al.	1996		√		√				√	√	√	5
Li et al.	2002		√						√	√	√	4
Li et al.	2016									√	√	2
Liu et al.	2020		√						√	√	√	4
Sun et al.	2020		√						√	√	√	4
Tang et al.	2006		√						√	√		3
Tang et al.	2010								√	√	√	3
Wang et al.	2019		√		√				√	√	√	5
Zhao et al.	2015								√	√	√	3
Zhao et al.	2018									√	√	2

(1) Random sequence generation; (2) baseline characteristics; (3) allocation concealment; (4) random housing; (5) blinding of trial caregivers; (6) random outcome assessment; (7) blinding of outcome assessment; (8) incomplete outcome data; (9) selective outcome reporting; and (10) other sources of bias.

Selection bias: 1–3; performance bias: 4, 5; detection bias: 6, 7; attrition bias: 8; reporting bias: 9; and other sources of bias: 10.

### Meta-analysis

#### Bone mineral density

**Figure 2.**
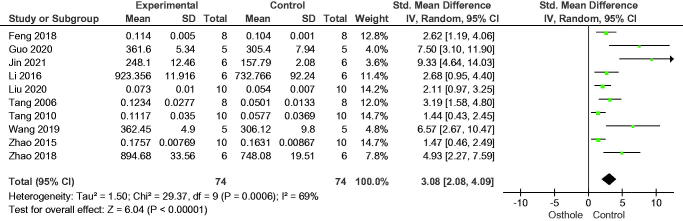
Forest plot comparing bone mineral density between osthole and control groups. SD: standard deviation; Std: standard.

This meta-analysis included ten trials. BMD was higher in the osthole treatment group than in the control group (SMD = 3.08, 95% CI = 2.08–4.09, *p* < 0.00001) (Figure 2). Subgroup analysis revealed a consistent increase in BMD across different doses and durations of osthole treatment (Table 3). The increase in BMD was greatest with osthole <10 mg/kg/day and duration of osthole treatment >2 months.

**Table 3.** Subgroup analysis of bone mineral density according to the dose and duration of osthole treatment.

Subgroup	Standardized mean difference (95% confidence interval)	I²	*p* value
Dose			
≤10 mg/kg	3.82 (2.57–5.07)	63	0.000
>10 mg/kg	1.46 (0.74–2.17)	0	0.000
Duration			
≤2 months	2.50 (1.44–3.56)	67	0.000
>2 months	4.78 (2.08–7.49)	74	0.000

#### Bone histomorphometric and biomechanical parameters

**Figure 3.**
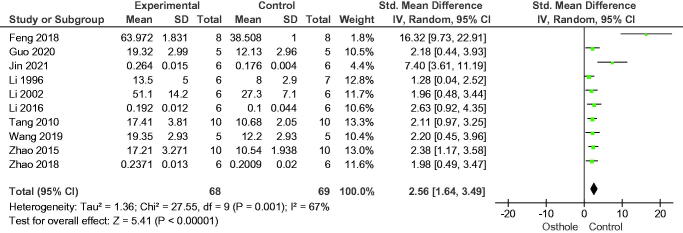
Forest plot comparing bone volume fraction between osthole and control groups. BV/TV: bone volume/tissue volume; SD: standard deviation; Std: standard.

**Figure 4.**
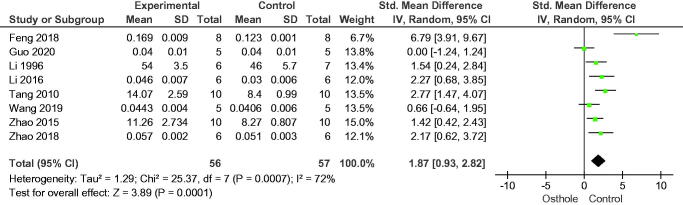
Forest plot comparing trabecular thickness between osthole and control groups. SD: standard deviation; Std: standard.

**Figure 5.**
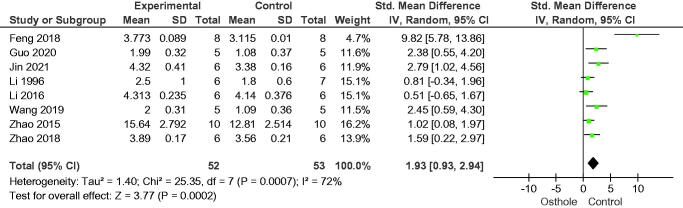
Forest plot comparing trabecular number between osthole and control groups. SD: standard deviation; Std: standard.

**Figure 6.**
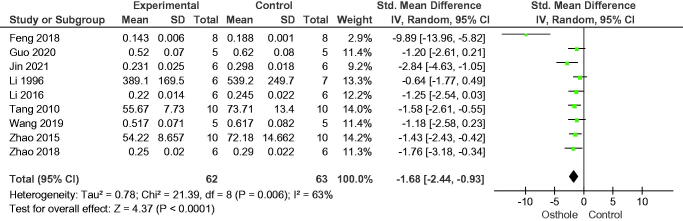
Forest plot comparing trabecular separation between osthole and control groups. SD: standard deviation; Std: standard.

In the 10 studies included in this analysis, osthole increased bone volume fraction (SMD = 2.56, 95% CI = 1.64–3.49, *p* < 0.00001), eight studies reported trabecular thickness (SMD = 1.87, 95% CI = 0.93–2.82, *p* = 0.0001), and eight studies reported trabecular number (SMD = 1.93, 95% CI = 0.93–2.94, *p* = 0.0002); nine studies reported osthole reduced trabecular separation (SMD = −1.68, 95% CI = −2.44 to −0.93, *p* < 0.0001) (Figures 3–6).

**Figure 7.**
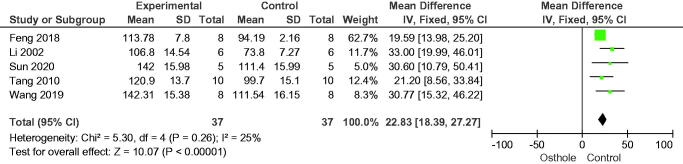
Forest plot comparing maximum load between osthole and control groups. SD: standard deviation.

**Figure 8.**
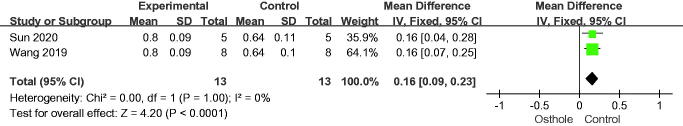
Forest plot comparing maximum deflection between osthole and control groups. SD: standard deviation.

**Figure 9.**
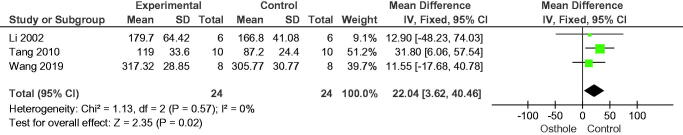
Forest plot comparing maximum stiffness between osthole and control groups. SD: standard deviation.

Three-point bending tests were performed on femoral shaft samples to measure bone biomechanical parameters. In comparison with the control group, osthole significantly increased the maximum load (mean difference [MD] = 22.83, 95% CI = 18.39–27.27, *p* < 0.00001) in five studies, maximum deflection (MD = 0.16, 95% CI = 0.09–0.23, *p* < 0.0001) in two studies, and maximum stiffness (MD = 22.04, 95% CI = 3.62–40.46, *p* = 0.02) in three studies (Figures 7–9).

#### Proliferation of osteoclasts

**Figure 10.**
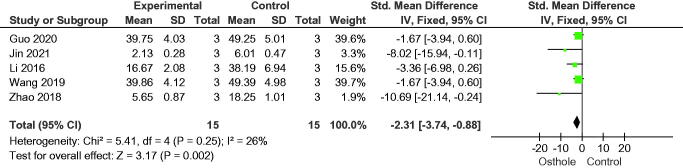
Forest plot comparing osteoclast numbers between osthole and control groups. SD: standard deviation; Std: standard.

**Figure 11.**
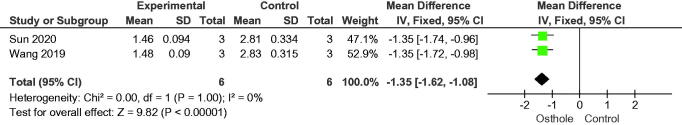
Forest plot comparing nuclear factor of activated T-cells 1 between osthole and control groups. SD: standard deviation.

**Figure 12.**
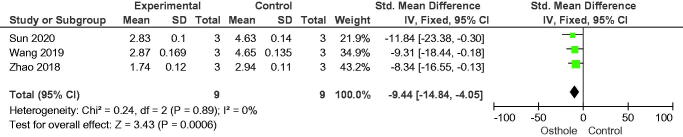
Forest plot comparing cathepsin K between osthole and control groups. SD: standard deviation; Std: standard.

**Figure 13.**
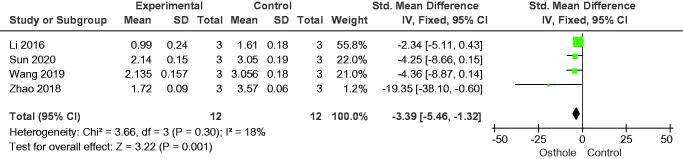
Forest plot comparing osteoclast-specific genes of tartrate-resistant acid phosphatase between osthole and control groups. SD: standard deviation; Std: standard.

**Figure 14.**
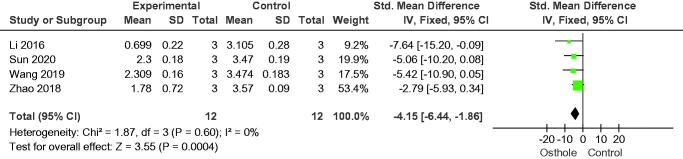
Forest plot comparing matrix metalloproteinase-9 between osthole and control groups. SD: standard deviation; Std: standard.

Five studies measured the number of osteoclasts in bone tissue and observed a higher number of bone osteoclasts in the control group than in the osthole group (SMD = −2.31, 95% CI = −3.74 to −0.88, *p* = 0.002) (Figure 10). In addition, osthole inhibited the expression of osteoclast-specific genes, including nuclear factor of activated T-cells 1, cathepsin K, tartrate-resistant acid phosphatase, and matrix metalloproteinase-9 (Figures 11–14).

#### Bone biochemical markers

**Figure 15.**
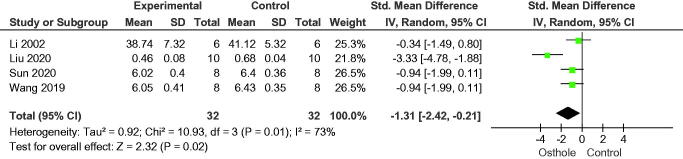
Forest plot comparing serum osteocalcin between osthole and control groups. SD: standard deviation; Std: standard.

**Figure 16.**
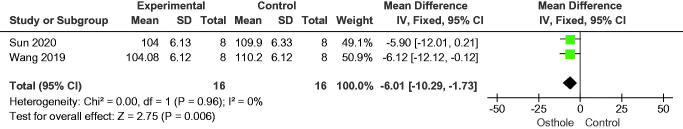
Forest plot comparing bone alkaline phosphatase between osthole and control groups. SD: standard deviation.

**Figure 17.**
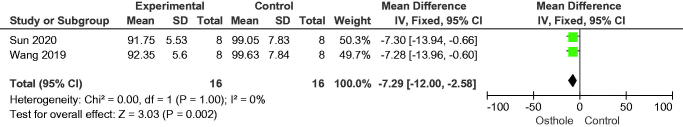
Forest plot comparing serum bone biochemical markers of tartrate-resistant acid phosphatase between osthole and control groups. SD: standard deviation.

Four studies analysed serum bone biochemical markers and observed lower osteocalcin (SMD = −1.31, 95% CI = −2.42 to −0.21, *p* = 0.02), two studies observed lower bone alkaline phosphatase (MD = −6.01, 95% CI = −10.29 to −1.73, *p* = 0.006), and lower tartrate-resistant acid phosphatase (MD = −7.29, 95% CI = −12.00 to −2.58, *p* = 0.002) in osthole groups than in control groups (Figures 15–17).

#### Sensitivity analysis and publication bias

**Figure 18.**
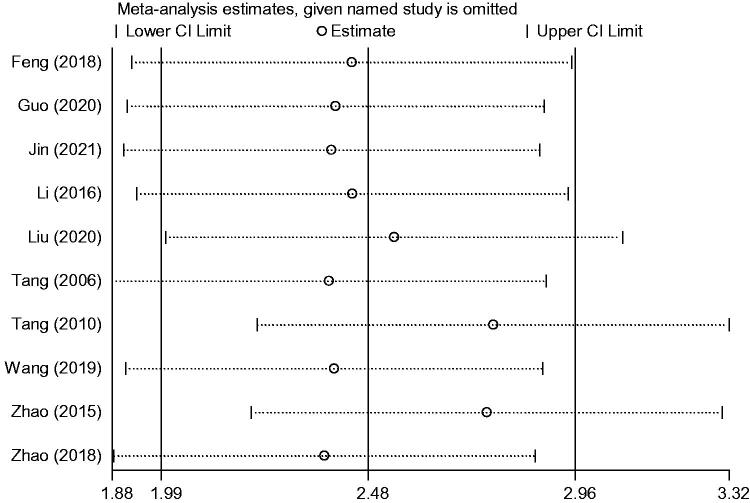
Sensitivity analysis of bone mineral density. CI: confidence interval.

Sensitivity analysis was performed using the method described above. The heterogeneity index and 95% CIs did not significantly change with exclusion of any study, indicating that the between-study differences were small and the results of this meta-analysis were robust (Figure 18). Because the included studies had approximately equal and small sample sizes, the outcomes data were presented as continuous variables. Therefore, funnel plots and Egger’s test were not used to assess publication bias, in accordance with guidance from Cochrane Collaboration.

## Discussion

This meta-analysis included animal experiments that evaluated the use of osthole in the treatment of osteoporosis. Osthole significantly increased BMD in osteoporotic rats. Subgroup analysis demonstrated that the increase in BMD varied with dose and duration of osthole treatment. The increase in BMD was greatest with osthole <10 mg/kg/day and duration of treatment >2 months.

Bone strength depends on bone mass and bone quality (Fonseca et al. 2014). BMD measured by dual-energy X-ray absorptiometry is the standard for diagnosis of osteoporosis and for determining treatment (Curry et al. 2018). BMD refers to the amount of bone mass per unit volume or area, i.e., the amount of bone tissue and bone matrix, osteoporosis is characterized by low BMD (Eastell et al. 2016). Trabecular bone volume fraction reflects the bone mass; trabecular thickness and number reflect the variation in bone mass. Trabecular separation represents the structure of trabeculae and is closely related to bone mass. Bone biomechanical parameters are the most direct indicators of bone quality and are used to assess the bone strength (Díaz et al. 2016). The results of this meta-analysis suggest that osthole improves the above-mentioned parameters and has a therapeutic effect on osteoporotic rats.

Bone remodelling is tightly regulated by bone-forming osteoblasts and bone-resorbing osteoclasts (Kim et al. 2020). When bone resorption is greater than bone formation, there is a net loss of bone tissue (Alippe et al. 2017), which can lead to osteoporosis. Osteoclasts are multinucleated cells derived from monocyte/macrophage lineage and are the only cells capable of bone resorption (Udagawa et al. 2021). The proliferation and differentiation of osteoclasts are regulated by cytokines, transcription factors and osteoclast-related genes such as macrophage-colony stimulating factor and tumour necrosis factor. The OPG/RANKL/RANK pathway is the critically important signalling pathway in the process (Meng et al. 2020). Receptor activator of nuclear factor kappa-Β ligand binds to receptors on the osteoclast surface; it regulates NF-κB signalling, activates nuclear factor of activated T-cells 1, and activates osteoclast-specific molecules (e.g., cathepsin K, matrix metalloproteinase-9, and tartrate-resistant acid phosphatase). Previous *in vitro* experiments demonstrated that osthole inhibits bone resorption by suppressing proliferation of osteoclasts and expression of osteoclast-specific genes (Zhai et al. 2014; Lv et al. 2016; Ma et al. 2019). The results of this meta-analysis confirmed that osthole had similar effects *in vivo*, presumably through increased expression of OPG/RANKL (Zhai et al. 2014; Li et al. 2016).

Most osteoporotic fractures occur in postmenopausal women (Black and Rosen 2016) because of decreased oestrogen levels that lead to reduced bone mass and microarchitectural degeneration. Ovariectomized rats have severe oestrogen deficiency, simulating postmenopausal osteoporosis; these rats are commonly used to model osteoporosis (Khosla et al. 2011). In postmenopausal osteoporosis, there is high bone turnover, as well as increased bone remodelling, resorption, and formation (Eastell and Szulc 2017). As for bone biochemical markers, only ovariectomized rats were included in the analysis for comparison. The result showed that osthole reduce bone metabolism markers (e.g., osteocalcin) in ovariectomized rats.

Numerous studies have been conducted regarding the effects of osthole on osteoblasts. Zhai et al. (2014) demonstrated increased osteoblast proliferation after 48 h of *in vitro* culture in different concentrations of osthole (0.1–50 μmol/L). Similar results were observed in other studies (Zhang et al. 2010). However, Ming et al. (2011) observed that osthole did not stimulate osteoblast proliferation *in vitro* at 0.1–100 μmol/L, even at 10–100 μmol/L, osthole inhibited osteoblast proliferation. Although the effects of osthole on osteoblast proliferation vary among studies, almost all studies have demonstrated that osthole can promote osteoblast differentiation (Tang et al. 2010; Gao et al. 2013; Zheng et al. 2018). For osthole could activate Wnt/β-catenin pathways and up-regulate BMP2 expression (Yu et al. 2020). Further *in vitro* and *in vivo* studies are needed to confirm the efficacy of osthole in the treatment of osteoporosis.

Importantly, some randomized controlled trials have evaluated the effects of osthole-loaded *N*-octyl-*O*-sulfonyl chitosan micelles on bone properties. Chitosan micelles, derived from animal shells, were used as carriers for osthole; they demonstrated superior effects on bone properties, compared with osthole (Wang et al. 2020).

As population ageing become a universal phenomenon in human society, the treatment of osteoporosis will be more important. Teriparatide is a rare bone-forming drug in the market which side effects and safety need to be further verified. And it is very expensive, so novel drugs that can promote bone formation are needed. In comparison with the other drugs used in the treatment of osteoporosis, osthole (a natural compound) has fewer side effects including drowsiness, nausea and stomach discomfort. According to the record in Chinese Pharmacopoeia, it has a limited degree of toxicity (Li et al. 2015). It can promote bone formation as well as inhibit bone absorption. Osthole is very cheap, just costs only $5.16/kg; therefore, it is suitable for the development of new drugs.

### Strengths and limitations

To our knowledge, this is the first meta-analysis of the treatment of osteoporotic rats with osthole. This meta-analysis included randomized controlled trials with high evidence and reliability levels, and can serve as a basis for future clinical studies. In addition, subgroup analyses were conducted to evaluate the effects of osthole dose and duration of treatment on BMD.

However, there were some limitations in this meta-analysis. Some included studies had flawed methodology and poor quality. All studies were conducted on a small sample of animals, and some outcomes were reported only in a few studies. Ovariectomized and senile osteoporosis models best simulate postmenopausal and senile osteoporosis, and future studies should evaluate the effects of osthole in both models.

## Conclusions

In this meta-analysis, we found that osthole increased BMD, improved bone parameters and osteoporosis, and inhibited osteoclast proliferation and expression of osteoclast-specific genes in experimental rats. With the clinical data in this study, we believe that osthole is a promising treatment for osteoporosis. Also experimental and clinical studies with larger samples are needed in the future to promote its application.

## References

[CIT0001] Alippe Y, Wang C, Ricci B, Xiao J, Qu C, Zou W, Novack DV, Abu-Amer Y, Civitelli R, Mbalaviele G. 2017. Bone matrix components activate the NLRP3 inflammasome and promote osteoclast differentiation. Sci Rep. 7(1):6630–6640. 28747793 10.1038/s41598-017-07014-0 PMC5529467

[CIT0002] Black DM, Rosen CJ. 2016. Clinical practice. Postmenopausal osteoporosis. N Engl J Med. 374(3):254–262. 26789873 10.1056/NEJMcp1513724

[CIT0003] Borgström F, Karlsson L, Ortsäter G, Norton N, Halbout P, Cooper C, Lorentzon M, McCloskey EV, Harvey NC, Javaid MK, et al. 2020. Fragility fractures in Europe: burden, management and opportunities. Arch Osteoporos. 15(1):59–79. 32306163 10.1007/s11657-020-0706-y PMC7166207

[CIT0004] Cosman F, de Beur SJ, LeBoff MS, Lewiecki EM, Tanner B, Randall S, Lindsay R. 2014. Clinician’s guide to prevention and treatment of osteoporosis. Osteoporos Int. 25(10):2359–2381. 25182228 10.1007/s00198-014-2794-2 PMC4176573

[CIT0005] Curry SJ, Krist AH, Owens DK, Barry MJ, Caughey AB, Davidson KW, Doubeni CA, Epling JJ, Kemper AR, Kubik M, et al. 2018. Screening for osteoporosis to prevent fractures: US preventive services task force recommendation statement. JAMA. 319(24):2521–2531. 29946735 10.1001/jama.2018.7498

[CIT0006] Díaz DH, Rodas JA, Bozzini CE, Mandalunis PM, Escudero ND. 2016. Sequential administration of alendronate and strontium ranelate: histomorphometry and bone biomechanics in ovariectomized animals. Acta Odontol Latinoam. 29(2):168–177. 27731487

[CIT0007] Eastell R, O’Neill TW, Hofbauer LC, Langdahl B, Reid IR, Gold DT, Cummings SR. 2016. Postmenopausal osteoporosis. Nat Rev Dis Primers. 2:16069–16084. 27681935 10.1038/nrdp.2016.69

[CIT0008] Eastell R, Szulc P. 2017. Use of bone turnover markers in postmenopausal osteoporosis. Lancet Diabetes Endocrinol. 5(11):908–923. 28689768 10.1016/S2213-8587(17)30184-5

[CIT0009] Feng X, He JP, Hua JR, LI H. 2018. The protective effect of osthole on bone loss induced by simulated microgravity. Chin J Osteoporos. 24:1074–1079.

[CIT0010] Fonseca H, Moreira-Gonçalves D, Coriolano HJ, Duarte JA. 2014. Bone quality: the determinants of bone strength and fragility. Sports Med. 44(1):37–53. 24092631 10.1007/s40279-013-0100-7

[CIT0011] Fuggle NR, Curtis EM, Ward KA, Harvey NC, Dennison EM, Cooper C. 2019. Fracture prediction, imaging and screening in osteoporosis. Nat Rev Endocrinol. 15(9):535–547. 31189982 10.1038/s41574-019-0220-8

[CIT0012] Gao LN, An Y, Lei M, Li B, Yang H, Lu H, Chen FM, Jin Y. 2013. The effect of the coumarin-like derivative osthole on the osteogenic properties of human periodontal ligament and jaw bone marrow mesenchymal stem cell sheets. Biomaterials. 34(38):9937–9951. 24095254 10.1016/j.biomaterials.2013.09.017

[CIT0013] Guo Y, Wang LN, Ma Y, Zheng SY. 2020. Effects of osthole-loaded *N*-octyl-*O*-sulfonyl chitosan micelles on bone microstructures and bone resorption in ovariectomized osteoporotic rats. Chin J Osteoporos. 26:1262–1267.

[CIT0014] Guzon-Illescas O, Perez FE, Crespí VN, Quirós DF, Peña M, Alonso-Blas C, García-Vadillo A, Mazzucchelli R. 2019. Mortality after osteoporotic hip fracture: incidence, trends, and associated factors. J Orthop Surg Res. 14(1):203–211. 31272470 10.1186/s13018-019-1226-6 PMC6610901

[CIT0015] Hernlund E, Svedbom A, Ivergård M, Compston J, Cooper C, Stenmark J, McCloskey EV, Jönsson B, Kanis JA. 2013. Osteoporosis in the European Union: medical management, epidemiology and economic burden. A report prepared in collaboration with the International Osteoporosis Foundation (IOF) and the European Federation of Pharmaceutical Industry Associations (EFPIA). Arch Osteoporos. 8(1–2):136–250. 24113837 10.1007/s11657-013-0136-1 PMC3880487

[CIT0016] Hooijmans CR, Rovers MM, de Vries RB, Leenaars M, Ritskes-Hoitinga M, Langendam MW. 2014. SYRCLE's risk of bias tool for animal studies. BMC Med Res Methodol. 14:43–51. 24667063 10.1186/1471-2288-14-43 PMC4230647

[CIT0017] Jin ZX, Liao XY, Da WW, Zhao YJ, Li XF, Tang DZ. 2021. Osthole enhances the bone mass of senile osteoporosis and stimulates the expression of osteoprotegerin by activating β-catenin signaling. Stem Cell Res Ther. 12(1):154–163. 33640026 10.1186/s13287-021-02228-6 PMC7912492

[CIT0018] Keshishi D, Makunts T, Abagyan R. 2021. Common osteoporosis drug associated with increased rates of depression and anxiety. Sci Rep. 11(1):23956–23963. 34907232 10.1038/s41598-021-03214-x PMC8671447

[CIT0019] Khan AA, Morrison A, Hanley DA, Felsenberg D, McCauley LK, O’Ryan F, Reid IR, Ruggiero SL, Taguchi A, Tetradis S, et al. 2015. Diagnosis and management of osteonecrosis of the jaw: a systematic review and international consensus. J Bone Miner Res. 30(1):3–23. 25414052 10.1002/jbmr.2405

[CIT0020] Khosla S, Melton LR, Riggs BL. 2011. The unitary model for estrogen deficiency and the pathogenesis of osteoporosis: is a revision needed? J Bone Miner Res. 26(3):441–451. 20928874 10.1002/jbmr.262 PMC3179298

[CIT0021] Kim JM, Lin C, Stavre Z, Greenblatt MB, Shim JH. 2020. Osteoblast-osteoclast communication and bone homeostasis. Cells. 9(9):2073–2086. 10.3390/cells9092073 PMC7564526 32927921

[CIT0022] Li X, Xue C, Wang L, Tang D, Huang J, Zhao Y, Chen Y, Zhao D, Shi Q, Wang Y, et al. 2016. Osteoprotective effects of osthole in a mouse model of 5/6 nephrectomy through inhibiting osteoclast formation. Mol Med Rep. 14(4):3769–3776. 27571745 10.3892/mmr.2016.5687

[CIT0023] Li XX, Hara I, Matsumiya T. 2002. Effects of osthole on postmenopausal osteoporosis using ovariectomized rats; comparison to the effects of estradiol. Biol Pharm Bull. 25(6):738–742. 12081139 10.1248/bpb.25.738

[CIT0024] Li YM, Jia M, Li HQ, Zhang ND, Wen X, Rahman K, Zhang QY, Qin LP. 2015. *Cnidium monnieri*: a review of traditional uses, phytochemical and ethnopharmacological properties. Am J Chin Med. 43(5):835–877. 26243582 10.1142/S0192415X15500500

[CIT0025] Li YY, Gao LJ, Zhang YX, Liu SJ, Cheng S, Liu YP, Jia CX. 2020. Bisphosphonates and risk of cancers: a systematic review and meta-analysis. Br J Cancer. 123(10):1570–1581. 32901134 10.1038/s41416-020-01043-9 PMC7652831

[CIT0026] Li ZY, Wu T, Li QN, Lin BY. 1996. Quantitative study on the effect of osthole on proximal tibiae in ovariectomized rats. Acta Pharm Sin. 31:327–332. 9275709

[CIT0027] Liu Y, Wang LH, Yi F. 2020. Study on the effect of osthole on ovariectomized osteoporotic rats based on the expression of BGP and TGF-β1. Chin Med Mode Dis Edu China. 18:99–101.

[CIT0028] Lv S, Zhang Y, Yan M, Mao H, Pan C, Gan M, Fan J, Wang G. 2016. Inhibition of osteolysis after local administration of osthole in a TCP particles-induced osteolysis model. Int Orthop. 40(7):1545–1552. 26498175 10.1007/s00264-015-3021-2

[CIT0029] Ma Y, Wang L, Zheng S, Xu J, Pan Y, Tu P, Sun J, Guo Y. 2019. Osthole inhibits osteoclasts formation and bone resorption by regulating NF-κB signaling and NFATc1 activations stimulated by RANKL. J Cell Biochem. 120(9):16052–16061. 31081953 10.1002/jcb.28886

[CIT0030] Meng B, Wu D, Cheng Y, Huang P, Liu Y, Gan L, Liu C, Cao Y. 2020. Interleukin-20 differentially regulates bone mesenchymal stem cell activities in RANKL-induced osteoclastogenesis through the OPG/RANKL/RANK axis and the NF-κB, MAPK and AKT signalling pathways. Scand J Immunol. 91(5):e12874-12885. 32090353 10.1111/sji.12874

[CIT0031] Ming LG, Zhou J, Cheng GZ, Ma HP, Chen KM. 2011. Osthol, a coumarin isolated from common *Cnidium* fruit, enhances the differentiation and maturation of osteoblasts *in vitro*. Pharmacology. 88(1-2):33–43. 21734431 10.1159/000328776

[CIT0032] Minisola S, Cipriani C, Grotta GD, Colangelo L, Occhiuto M, Biondi P, Sonato C, Vigna E, Cilli M, Pepe J. 2019. Update on the safety and efficacy of teriparatide in the treatment of osteoporosis. Ther Adv Musculoskelet Dis. 11:1759720X–19877994X. 10.1177/1759720X19877994 PMC6778993 31632472

[CIT0033] Moher D, Liberati A, Tetzlaff J, Altman DG. 2009. Preferred reporting items for systematic reviews and meta-analyses: the PRISMA statement. Ann Intern Med. 151(4):264–269. 19622511 10.7326/0003-4819-151-4-200908180-00135

[CIT0034] Si L, Winzenberg TM, Jiang Q, Chen M, Palmer AJ. 2015. Projection of osteoporosis-related fractures and costs in China: 2010–2050. Osteoporos Int. 26(7):1929–1937. 25761729 10.1007/s00198-015-3093-2

[CIT0035] Sun J, Guo Y, Ma Y, Wang LN. 2020. Effects of osthole-loaded *N*-octyl-*O*-sulfonyl chitosan micelles on bone metabolism and biomechanical properties in osteoporotic rats. Chin Arch of Trad Chin Med. 39:126–129.

[CIT0036] Sun Y, Yang A, Lenon GB. 2020. Phytochemistry, ethnopharmacology, pharmacokinetics and toxicology of *Cnidium monnieri* (L.) Cusson. Int J Mol Sci. 21(3):1006–1056. 10.3390/ijms21031006 PMC7037677 32028721

[CIT0037] Tang DZ, Hou W, Zhou Q, Zhang M, Holz J, Sheu TJ, Li TF, Cheng SD, Shi Q, Harris SE, et al. 2010. Osthole stimulates osteoblast differentiation and bone formation by activation of beta-catenin-BMP signaling. J Bone Miner Res. 25(6):1234–1245. 20200936 10.1002/jbmr.21 PMC3153131

[CIT0038] Tang QF, Kong LJ, Gu ZL, Xie ML. 2006. Experimental study on the inhibition of osthole on osteoporosis in rats. Chin Trad Herb Drugs. 11:1700–1702.

[CIT0039] Udagawa N, Koide M, Nakamura M, Nakamichi Y, Yamashita T, Uehara S, Kobayashi Y, Furuya Y, Yasuda H, Fukuda C, et al. 2021. Osteoclast differentiation by RANKL and OPG signaling pathways. J Bone Miner Metab. 39(1):19–26. 33079279 10.1007/s00774-020-01162-6

[CIT0040] Wang L, Yu W, Yin X, Cui L, Tang S, Jiang N, Cui L, Zhao N, Lin Q, Chen L, et al. 2021. Prevalence of osteoporosis and fracture in China: the China osteoporosis prevalence study. JAMA Netw Open. 4(8):e2121106–2121117. 34398202 10.1001/jamanetworkopen.2021.21106 PMC8369359

[CIT0041] Wang L, Zheng S, Huang G, Sun J, Pan Y, Si Y, Tu P, Xu G, Ma Y, Guo Y. 2020. Osthole-loaded *N*-octyl-*O*-sulfonyl chitosan micelles (NSC-OST) inhibits RANKL-induced osteoclastogenesis and prevents ovariectomy-induced bone loss in rats. J Cell Mol Med. 24(7):4105–4117. 32126148 10.1111/jcmm.15064 PMC7171421

[CIT0042] Yu H, Zhu D, Liu P, Yang Q, Gao J, Huang Y, Chen Y, Gao Y, Zhang C. 2020. Osthole stimulates bone formation, drives vascularization and retards adipogenesis to alleviate alcohol-induced osteonecrosis of the femoral head. J Cell Mol Med. 24(8):4439–4451. 32135036 10.1111/jcmm.15103 PMC7176840

[CIT0043] Yuan F, Peng W, Yang C, Zheng J. 2019. Teriparatide versus bisphosphonates for treatment of postmenopausal osteoporosis: a meta-analysis. Int J Surg. 66:1–11. 30890377 10.1016/j.ijsu.2019.03.004

[CIT0044] Zhai YK, Pan YL, Niu YB, Li CR, Wu XL, Fan WT, Lu TL, Mei QB, Xian CJ. 2014. The importance of the prenyl group in the activities of osthole in enhancing bone formation and inhibiting bone resorption *in vitro*. Int J Endocrinol. 2014:921954–921969. 25147567 10.1155/2014/921954 PMC4131490

[CIT0045] Zhang W, Ma D, Zhao Q, Ishida T. 2010. The effect of the major components of Fructus Cnidii on osteoblasts *in vitro*. J Acupunct Meridian Stud. 3(1):32–37. 20633513 10.1016/S2005-2901(10)60005-2

[CIT0046] Zhang ZR, Leung WN, Li G, Kong SK, Lu X, Wong YM, Chan CW. 2017. Osthole enhances osteogenesis in osteoblasts by elevating transcription factor osterix via cAMP/CREB signaling *in vitro* and *in vivo*. Nutrients. 9(6):588–602. 10.3390/nu9060588 PMC5490567 28629115

[CIT0047] Zhao D, Wang Q, Zhao Y, Zhang H, Sha N, Tang D, Liu S, Lu S, Shi Q, Zhang Y, et al. 2018. The naturally derived small compound osthole inhibits osteoclastogenesis to prevent ovariectomy-induced bone loss in mice. Menopause. 25(12):1459–1469. 29944638 10.1097/GME.0000000000001150

[CIT0048] Zhao YJ, Tang DZ, Chen SD, Zheng WC. 2015. Comparison study of the effect of different doses of osthole on OPG knockout mice and OVX rats. Chin J Osteoporos. 21:147–151.

[CIT0049] Zheng S, Ma Y, Guo Y, Wang L, Pan Y. 2018. Osthole promote differentiation and inhibit proliferation of osteoblast by activating Wnt signaling and endoplasmic reticulum stress. Pharmacogn Mag. 14:58–63.29576702

